# Effect of Oral Losartan on Orthobiologics: Implications for Platelet-Rich Plasma and Bone Marrow Concentrate—A Rabbit Study

**DOI:** 10.3390/ijms21197374

**Published:** 2020-10-06

**Authors:** Gilberto Y. Nakama, Sabrina Gonzalez, Polina Matre, Xiaodong Mu, Kaitlyn E. Whitney, Hajime Utsunomiya, Justin W. Arner, Marc J. Philippon, Sudheer Ravuri, Johnny Huard

**Affiliations:** 1 Center for Regenerative Sports Medicine, Steadman Philippon Research Institute, Vail, CO 81657, USA; gilbertoyn@gmail.com (G.Y.N.); smgonzalez95@gmail.com (S.G.); kwhitney@thesteadmanclinic.com (K.E.W.); hajime.utsu@gmail.com (H.U.); sravuri@sprivail.org (S.R.); 2 Department of Orthopaedic Surgery, University of Texas Health Science Center at Houston, Houston, TX 77054, USA; pmatre@hotmail.com (P.M.); xiaodong_m@yahoo.com (X.M.); 3 The Steadman Clinic, Vail, CO 81657, USA; jarner@thesteadmanclinic.com (J.W.A.); drphilippon@sprivail.org (M.J.P.)

**Keywords:** fibrosis, losartan, transforming growth factor-1 beta (TGF-β1), bone marrow concentrate (BMC), leukocyte-poor platelet-rich plasma (LP-PRP)

## Abstract

Recent efforts have focused on customizing orthobiologics, such as platelet-rich plasma (PRP) and bone marrow concentrate (BMC), to improve tissue repair. We hypothesized that oral losartan (a TGF-β1 blocker with anti-fibrotic properties) could decrease TGF-β1 levels in leukocyte-poor PRP (LP-PRP) and fibrocytes in BMC. Ten rabbits were randomized into two groups (N = 5/group): osteochondral defect + microfracture (control, group 1) and osteochondral defect + microfracture + losartan (losartan, group 2). For group 2, a dose of 10mg/kg/day of losartan was administrated orally for 12 weeks post-operatively. After 12 weeks, whole blood (WB) and bone marrow aspirate (BMA) samples were collected to process LP-PRP and BMC. TGF-β1 concentrations were measured in WB and LP-PRP with multiplex immunoassay. BMC cell populations were analyzed by flow cytometry with CD31, CD44, CD45, CD34, CD146 and CD90 antibodies. There was no significant difference in TGF-β1 levels between the losartan and control group in WB or LP-PRP. In BMC, the percentage of CD31+ cells (endothelial cells) in the losartan group was significantly higher than the control group (*p* = 0.008), while the percentage of CD45+ cells (hematopoietic cells-fibrocytes) in the losartan group was significantly lower than the control group (*p* = 0.03).

## 1. Introduction

Fibrosis is the excessive formation of connective tissue as a result of exaggerated deposition of extracellular matrix (ECM) factors (i.e., collagen) during tissue healing [1,2,3]. Fibrotic tissue stiffens a variety of tissues and organs, including cardiac muscle, skeletal muscle, lung, liver, kidney and cartilage, decreasing their function while predisposing those tissues for further injury [1,2,3,4,5]. After anterior cruciate ligament reconstruction of the knee, arthrofibrosis is an undesirable complication characterized by increased fibroblast infiltration in the joint that causes pain, decreases range of motion, and impairs joint function [6]. As an example of fibrotic impairment of cardiac muscle, the lack of hypertension control causes myocardial injury, and the perpetuation of injury leads to pathological myocardial repair that results in the recruitment of fibroblasts [7,8]. The main sources of myocardial fibrous tissue are resident fibroblasts, circulating bone marrow-derived fibrocytes, and fibroblasts from the endothelial mesenchymal transition (EndMT) [7,8]. The EndMT pathway plays a key role in cytoskeleton remodeling and scar tissue formation through the activation of endothelial cells (CD31+ cells) that undergo phenotypical changes, leading to the breakdown of basement membranes and reaching interstitial tissues [9,10,11]. Transforming growth factor-beta 1 (TGF-β1) plays a key role in EndMT activation and proliferation through the TGF-β1/Smad-dependent pathway that regulates TGF-β1 transcription, collagen expression [7,8]. The TGF-β1/Smad-dependent pathway is modulated by renin-angiotensin system through angiotensin II [8,12,13]. Thus, inhibition of the TGF-β1/Smad-dependent pathway using targeted agents may be a promising strategy to downregulate EndMT and, ultimately, fibrosis formation in musculoskeletal tissue. 

There is increasing interest in losartan (Cozaar^®^), an anti-hypertensive medication and selective angiotensin II type 1 (AT1) receptor antagonist with antifibrotic properties, to improve skeletal muscle and cartilage repair, and to prevent arthrofibrosis [12,13,14,15,16]. Although losartan’s main mechanism of activation is to block the binding of angiotensin II to the AT 1 receptor [17], losartan also exerts effects on EndMT. In fact, Wu et al. [8] observed that spontaneous hypertensive rats treated with losartan for 12 weeks inhibited TGF-β/Smad signaling, EndMT and myocardial fibrosis. In this study, the suppression of EndMT was observed through an increase in CD31+ cells (endothelial cells) and a decrease in fibroblast-specific protein 1 through immunofluorescence in myocardial tissue [8]. Bartko et al. [18] also reported that oral losartan significantly reduced CD45+ cells (hematopoietic cell marker), and concluded that these CD45+ cells represent fibrocytes recruited by TGF-β mediated endothelial activation after myocardial infarction in sheep. 

The role of TGF-β1 neutralization blockers, such as losartan, has been extensively evaluated in damaged skeletal muscle conditions [12,14,15,19]. Oral losartan combined with platelet-rich plasma (PRP) therapy has also effectively reduced skeletal muscle fibrosis and subsequently improved the therapeutic efficacy of PRP for muscle healing [20]. Decreasing TGF-β1 expression and fibrocyte concentrations in orthobiologic agents, such as leukocyte-poor platelet-rich plasma (LP-PRP) and bone marrow concentrate (BMC), may improve their biological efficacy and also enhance tissue healing after arthroscopic surgery. Therefore, the purpose of this study was to determine the systemic effect of oral losartan, an anti-fibrotic agent, on TGF-β1 in LP-PRP and cell compositions in BMC. We hypothesized that oral administration of losartan would influence biological factors and cellular concentrations in LP-PRP and BMC in an osteochondral injury and repair model. More specifically, we hypothesized that losartan would lower expression of transforming growth factor TGF-β1 in PRP, and BMC with higher percentage of CD31+ (endothelial cells) and lower percentage of CD45+ (hematopoietic cells–fibrocytes). 

## 2. Results

### 2.1. Whole Blood Complete Blood Count Results

The comparison between the losartan group and control group regarding the baseline complete blood count (CBC) of whole blood (WB) was shown in Table 1. There were no significant differences in red blood cell, white blood cell and platelet values between the two groups. 

### 2.2. LP-PRP Complete Blood Count and TGF-β1 Results

Considering control and losartan groups together, the number of platelets in LP-PRP was significantly higher than WB (*p* < 0.01); results shown in Table 2. The expression level of TGF-β1 was also significantly higher in LP-PRP than in WB (*p* = 0.01). Red blood cell and white blood cell values were not statistically different amongst the two groups.

### 2.3. Comparison of TGF-β1 Expression Level in Whole Blood and LP-PRP

**Figure 1:**
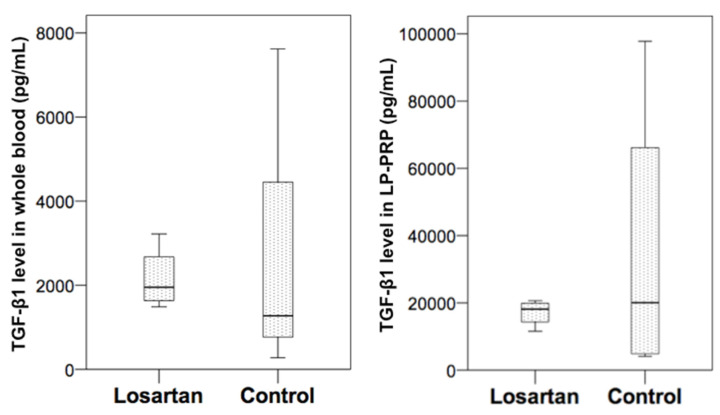
Losartan and control group comparison of transforming growth factor-beta 1 (TGF-β1) levels in whole blood (WB) and leukocyte-poor platelet-rich plasma (LP-PRP).

There was no significant difference regarding TGF-β1 levels between the losartan and control groups in WB and LP-PRP (losartan group vs. control group, pg/mL, WB: 1953.5 (1630.5–2677.0) vs. 1273.5 (766.0–4454.5), *p* = 0.69, LP-PRP: 18122.0 (14306.5–19904.0) vs. 20064.0 (4859.0–66129.0), *p* = 1.00). The interquartile range (IQR) of the losartan group was smaller than that of the control group (Figure 1). 

### 2.4. Bone Marrow Aspirate Complete Blood Count Results

The comparison between the losartan group and control group regarding the baseline CBC of bone marrow aspirate was shown in Table 3. There were no significant differences in red blood cell, white blood cell and platelet values between the two groups.

### 2.5. Bone Marrow Concentrate Complete Blood Count Results

The comparison between losartan group and control group regarding the baseline CBC of bone marrow concentrate was shown in Table 4. There were no significant differences in red blood cell and white blood cell values between the two groups. Platelet values of the control group were higher compared to losartan group (*p* = 0.04).

### 2.6. Bone Marrow Concentrate Flow Cytometry Results

**Figure 2:**
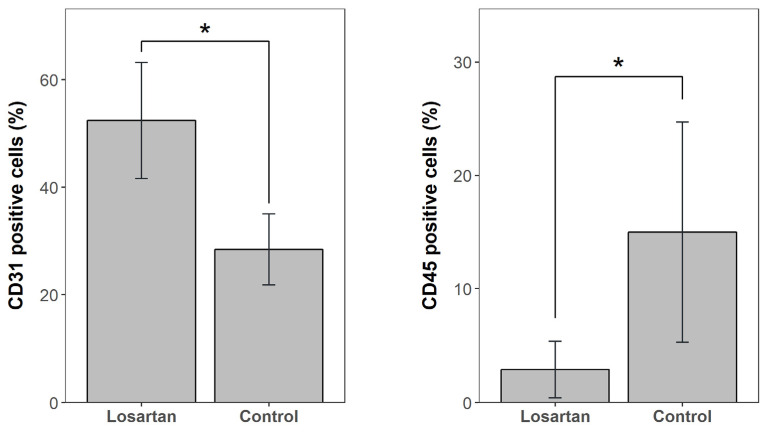
CD31+ cells and CD45+ cells in bone marrow concentrate (BMC) comparing the losartan group to the control group. * *p* < 0.05.

**Figure 3:**
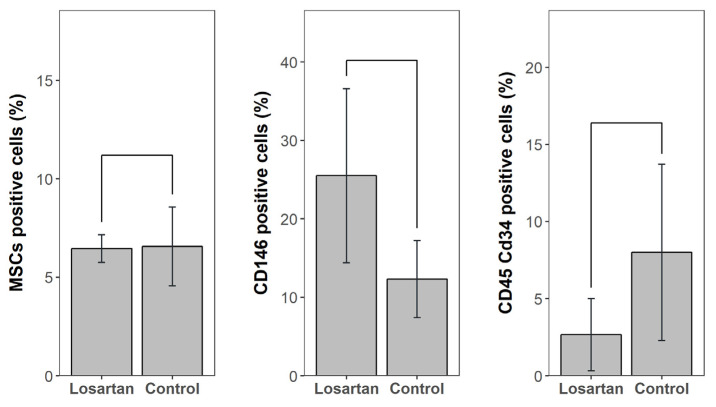
Mesenchymal stem cells (MSCs), CD146+ cells and CD45+ CD34+ cells in bone marrow concentrate (BMC) comparing the losartan group to the control group. Results were not statistically different between groups.

In BMC, the percentage of CD31+ cells in the losartan group was significantly higher than the control group (52.4 ± 10.8% vs. 28.4 ± 6.6%, *p* = 0.008), while the percentage of CD45+ cells in the losartan group was significantly lower than the control group (2.9 ± 2.5% vs. 15.0 ± 9.7%, *p* = 0.03) (Figure 2). We found no significant difference between the losartan group versus the control group in mesenchymal stem cells (MSCs) (6.45 ± 0.7% vs. 6.56 ± 2.0%, *p* = 0.31), CD146+ cells (25.5 ± 11.1% vs. 12.3 ± 4.9%, *p* = 0.15) and CD45+ CD34+ cells (2.66 ± 2.34% vs. 8.00 ± 5.71%, *p* = 0.056) (Figure 3). 

## 3. Discussion

The most important findings of this study were that oral losartan administration resulted in a significantly higher percentage of CD31+ cells and lower percentage of CD45+ cells in BMC. Additionally, TGF-β1 levels in the losartan group were not significantly lower in WB or LP-PRP compared to the control group. To the author’s knowledge, no other study has assessed the effect of oral losartan on BMC, specifically bone marrow cells. 

Bone marrow concentrate (BMC) and platelet-rich plasma (PRP) are biologic products derived, respectively, from bone marrow aspirate and WB, developed to concentrate various growth factors and bioactive molecules for healing enhancement. For over a decade, PRP has been used in orthopedics, dermatology and dentistry to enhance tissue healing [21,22,23,24]. Platelet-rich fibrin is another blood product, less prone to processing mistakes compared to platelet-rich plasma, since its preparation does not need anticoagulants [25]. Although platelet-rich fibrin tends to have a more replicable process than platelet-rich plasma, platelet-rich fibrin provides a higher white blood cell concentration than platelet-rich plasma, then we opted to use leukocyte-poor PRP to obtain lower leukocyte concentration to decrease the chance of inducing excessive inflammation after intra-articular administration [26,27,28]. Bone marrow is another biological product that has been used to treat musculoskeletal, cardiovascular, urinary, respiratory, digestive, integumentary and central nervous pathologies because of the potential to repair injured tissue [29,30,31] and has shown promising outcomes for various orthopedic conditions, including osteochondral defect and osteoarthritis [21,32,33,34]. 

Our findings suggest that losartan partially blocked the EndMT by increasing CD31+ cells (endothelial cells) and decreasing the expression of CD45+ cells (hematopoietic cells) measured in BMC, after stimulation induced by osteochondral defect and microfracture. In injured cardiac tissue, previous studies assessed the effect of losartan on EndMT and also observed an increase in CD31+ cells [8], and a decrease in CD45+ cells [18]. Zhu et al. [35] noticed a decrease in CD31 expression in the development of pulmonary vein stenosis of piglets submitted to pulmonary vein banding and conjectured that the turbulent flow could harm the endothelium, and that losartan could maintain the endothelium integrity through a positive interaction with vascular endothelial (VE)-cadherin. Our results corroborate with Terada et al. [13] who observed higher expression of CD31 (endothelial cells) at the muscle injury site in mice treated with PRP combined with oral losartan compared to control (no treatment), PRP and losartan alone. It has been previously reported that fibrocytes express CD45, a hematopoietic cell marker [18,36,37]. Bartko et al. [18] hypothesized that circulating bone marrow-derived cells differentiated into CD45+ cells (fibrocytes) at the injury site and observed that losartan blocked the EndMT on mitral valve endothelium after myocardial infarction showing lower expression of CD45+ cells. In summary, our results encourage new studies to administer losartan pre-operatively prior to harvesting bone marrow with the objective to enhance healing and postoperatively reduce fibrosis in patients who have a predisposition to develop arthrofibrosis or for patients undergoing cartilage repair procedures.

Interestingly, we did not observe significant differences between the losartan and control groups in LP-PRP or WB TGF-β1 concentrations. TGF-β1 is an essential Smad factor involved in downstream intracellular signaling and, more specifically, increasing ECM protein expression (i.e., matrix metalloproteinases [MMPs], tissue inhibitors of metalloproteinases [TIMPs]) [38]. The most notable role of TGF-β1 is stimulating fibrosis formation during connective tissue remodeling [39]. As a result, several anti-fibrotic medications have been developed to reduce local and systemic TGF-β1 levels by implicating the Smad2/3 pathway. Angiotensin-receptor blocker, losartan, has demonstrated its anti-fibrotic effects in cardiac [8,38,40,41,42] and skeletal muscle [12,13,14,15,19], as well as reducing systemic levels of TGF-β1 in transplant patients [43,44]. In the present study, there were no significant differences between TGF-β1 levels in the losartan and control groups; however, losartan dosing and timing in which the blood specimens were collected could have had an impact on TGF-β1 expression. Furthermore, losartan may not have an effect on TGF-β1 expression systemically in a rabbit model, but it is important to note that oral losartan treatment blocks the effect of TGF-β1 in synovial tissue, and improves cartilage repair, as described in our main study [44]. This is the first attempt at measuring and comparing TGF-β1 levels in WB and LP-PRP following losartan treatment in a rabbit model, but further evaluation of losartan, using a similar dosing regime, is necessary to clearly understand its effect on systemic TGF-β1 expression and, ultimately, customize LP-PRP for various musculoskeletal applications. 

The authors acknowledge that there were limitations in this study and have described these limitations in the following: The first limitation is that LP-PRP and BMC were derived from rabbits, and not from humans. TGF-β1 levels were detectable but we did not observe a difference between the groups in whole blood and LP-PRP. Some reasons to support this observation could be attributed to unavailability of specific rabbit TGF-β1 kits and issues related to losartan (dose response, preparation, frequency, delivery and time point evaluations). Although these findings suggest that oral intake of losartan (10 mg/kg/day) can be useful to modify the percentage of CD31+ cells and CD45+ cells in rabbit BMC reducing the incidence of fibrosis, the dose for humans must be clarified since oral losartan can induce hypotension in patients with normal arterial pressure, and the losartan dose assessed in this study is much higher than the usual therapeutic dose to control hypertension in humans. For future studies with this therapeutic approach, patients with normal arterial pressure must have the arterial pressure monitored after oral losartan administration, before the start of the treatment. Furthermore, patients that develop hypotension should be monitored closely and characteristics attributing to hypotension should be investigated.

## 4. Material and Methods

All animals were housed at the Center for Laboratory Animal Medicine and Care at the University of Texas Health Science Center at Houston and maintained according to approved Institutional Animal Care and Use Committee protocol (project identification code: AWC-17-0022, date of approval: 04/05/2017). The present study is an arm of a different study recently published that observed the effect of TGF-β1 blocking with losartan oral administration on rabbit cartilage repair after the creation of an osteochondral defect [45]. An osteochondral defect (5 mm in diameter and 2mm in depth) was made in the patellar groove of 10 New Zealand white rabbits that were divided into 2 groups (N = 5/group) randomly: a control group (osteochondral defect + microfracture, group 1) and losartan treated group (osteochondral defect + microfracture + losartan, group 2). More details of the surgical procedure are described in the main study [45]. A dose of 10mg/kg/day of losartan (Cozzar^®^, Merck, Readington, NJ, USA), the equivalent treatment dose in a mouse muscle injury model [12], was administrated orally to the rabbits in group 2 starting post-op day one through the day of euthanasia (12 weeks). The losartan dose was calculated based on previous literature that tested losartan in a mouse muscle injury model [12]. Rabbits were sacrificed after 12 weeks of losartan use. 

LP-PRP was tested since lower concentrations of leukocytes is less likely to induce excessive inflammation and fibrosis after an LP-PRP intra-articular injection [21,46,47,48].

### 4.1. LP-PRP: Collection and Processing

**Figure 4:**
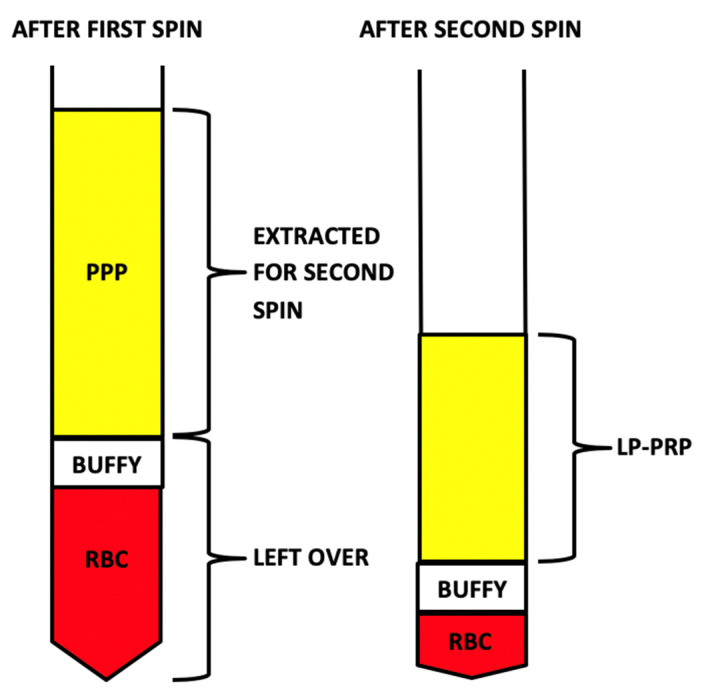
Demonstration of the peripheral blood separation and resulting layers after the first and second centrifugation steps. Leukocyte-poor platelet-rich plasma (LP-PRP); platelet-poor plasma (PPP); red blood cells (RBCs).

Whole blood (WB) was harvested shortly after euthanasia via cardiac puncture of the left ventricle through an open thoracotomy. For euthanasia, Pentobarbital Sodium 390 mg/mL was administered intravenously at 1 mL/4.5 kg. WB was collected in plastic vacutainers (BD, Franklin Lakes, NJ, USA) containing anticoagulant citrate dextrose formula-A (ACD-A) (Fenwal Laboratories, Lake Zurich, IL, USA) using 1.5 mL of ACD-A per 10 mL of WB. WB samples were processed using a benchtop centrifuge (LW Scientific Centrifuge Ultra Select 8-Place 4-Speed ULC-08AS-1501, LW Scientific, Inc., Lawrenceville, GA, USA) to prepare LP-PRP (Table 5 and Figure 4).

### 4.2. Bone Marrow Concentrate: Collection and Processing

**Figure 5:**
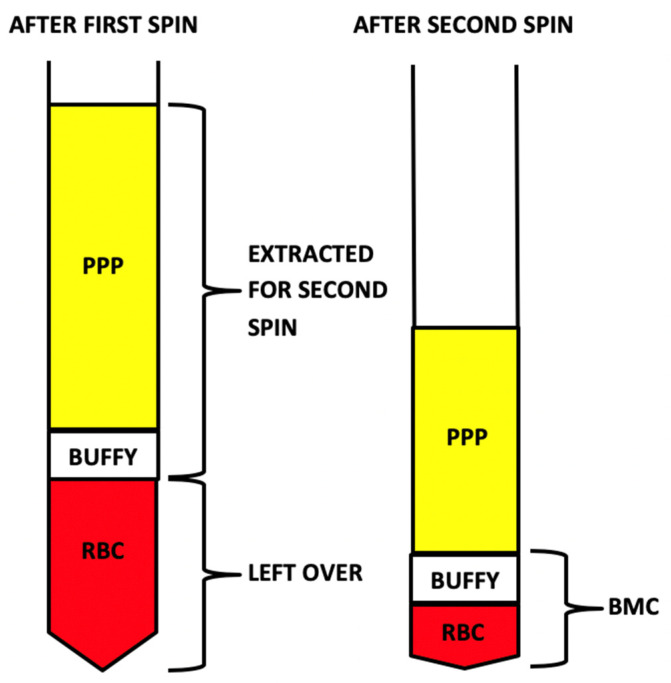
Demonstration of the bone marrow separation and resulting layers after the first and second centrifugation steps. Bone marrow concentrate (BMC); platelet-poor plasma (PPP); red blood cells (RBCs).

Bone marrow aspirate (BMA) was collected immediately after sacrifice by iliac crest aspiration. An incision was made to reach the rabbit iliac crest. Bone marrow samples were collected in plastic vacutainers (BD, Franklin Lakes, NJ, USA) containing ACD-A (Fenwal Laboratories, Lake Zurich, IL, USA) as an anti-coagulant (1.5 mL of ACD-A for 10 mL of BMA). Bone marrow samples were processed using a benchtop centrifuge (LW Scientific Centrifuge Ultra Select 8-Place 4-Speed ULC-08AS-1501, LW Scientific, Inc., Lawrenceville, GA, USA) to prepare LP-PRP and BMC **(**Table 5 and Figure 5). Filters were used for BMA before processing to BMC to remove spicules, micro blood clots and fat particles. 

### 4.3. Hematology Assessment

A standard hematology analyzer (IDEXX Procyte Dx, IDEXX, Westbrook, ME) was used to measure red blood cell, white blood cell and platelet values in whole blood (baseline), BMA (baseline), LP-PRP and BMC. Red blood cells were measured in M/uL (million per microliter), while white blood cells and platelets were measured in K/uL (thousand per microliter).

### 4.4. Whole Blood and LP-PRP Multiplex Assay and Analysis

Approximately 200 uL of WB and LP-PRP were pipetted into microcentrifuge tubes for Luminex^®^ multiplex immunoassays (EMD Millipore Corp, Billerca, MA, USA). Mix-species TGF-β magnetic bead panel (EMD Millipore Corp, Billerca, MA, USA) used were to measure TGF-β1 concentrations. A standard manufacturer’s protocol for the Luminex^®^ 200 system (Luminex Corp, Austin, TX, USA) multiplex instrument was utilized as previously published [47]. All reagents were prepared and stored according to manufacturer’s instructions. Briefly, background, standards, and controls were added in duplicate to the appropriate wells with serum matrix solution. The unknown samples were subsequently added in duplicate, along with premixed growth factor, cytokine and chemokine antibody immobilized magnetic beads. The plate was sealed and covered with foil during incubation with agitation at 600 RPM. Using a handheld magnet, the plate was washed two times using the 1X wash buffer provided. Antibodies were added to the plate and incubated at room temperature for 30 min at 600 RPM. Streptavidin-phycoerythrin solution was added and incubated at room temperature for 30 min at 600 RPM. Following two plate washes, drive fluid was added to re-suspend the beads at 300 RPM for five minutes. Finally, the plate was analyzed with the Luminex^®^ 200 xPONENT 3.1 system (Luminex Corp, Austin, TX, USA) using the xPonent^®^ software (EMD Millipore Corp, Billerica, MA, USA), which created a standard curve for each respective analyte utilizing a five-parameter logistic curve-fitting method with the median fluorescent intensity. TGF-β1 concentrations in the unknown samples were calculated using the five-parameter standard curve. 

### 4.5. Bone Marrow Flow Cytometry and Mononuclear Cell Isolation 

#### 4.5.1. Flow Cytometry Analysis

Cell populations were detected and measured by flow cytometry with the following antibodies: CD31, CD44 (Novus Biologicals, Littleton, CO, USA), CD45 (Biorad, Hercules, CA, USA), CD34 (Genetex, Irvine, CA, USA), CD146 (TermoFisher, Waltham, MA, USA) and CD90 (Abcam, Cambridge, MA, USA). Staining occurred after exclusion of nonviable cells by diamidino-2-phenylindole (Sigma-Aldrich, St. Louis, MO, USA). Flow cytometry analyses were performed using LSRII (BD, Franklin Lakes, NJ, USA) and Gallios Flow Cytometer, and data were analyzed by FlowJo software (BD, Franklin Lakes, NJ, USA). This analysis was performed on all BMC samples. 

#### 4.5.2. Mononuclear Cell Isolation by Density Gradient Centrifugation

Bone marrow cells from each rabbit were separated using lymphocyte separation Medium D = 1.080 g/mL (Corning, Corning, NY, USA) using density gradient centrifugation. Bone marrow cells were resuspended in 20 mL of PBS and carefully layered upon 10 mL of Ficoll separation medium. Ficoll gradients were centrifuged for 30 min at 300 g without break. Then, the bone marrow mononuclear cell layer was collected and washed in PBS. ACK (ammonium–chloride–potassium) lysing buffer (ThermoFisher, Waltham, MA, USA) was used for the lysis of red blood cells according to manufacturer’s instructions. Next, the bone marrow mononuclear cells were resuspended in PBS and prepared for flow cytometry analysis. 

### 4.6. Statistical Analysis

All data were reported in median and interquartile range (IQR) or mean ± SD. A Mann–Whitney U-test was used to compare groups. Wilcoxon signed rank test was performed for the comparison in the same specimen. Statistical analyses were performed using SPSS (version 21, SPSS Inc., Chicago, IL, USA) software package. The level of significance was set at a probability value of *p* < 0.05. A power analysis was performed using CD31 data from the first 6 rabbits (3 rabbits from each group). Assuming that dependent groups were compared using the Mann–Whitney U-test and with alpha set as 0.05, the effect size was calculated as d = 3.24, and 3 rabbits from each group were determined to reach to the power of 0.80. It was determined that five rabbits from each group would give the power of 0.99.

## 5. Conclusions

Oral losartan (10 mg/kg/day) did not significantly reduce TGF-β1 levels in WB or LP-PRP. However, oral losartan resulted in a significantly higher percentage of CD31+ cells and lower percentage of CD45+ cells in BMC. 

## Figures and Tables

**Table 1:** Whole blood (WB) comparison between control and losartan groups in complete blood count (CBC) values. Red blood cells were measured in M/uL, while white blood cells and platelets were measured in K/uL.

	Control		Losartan		*p* Value
	Median	IQR	Median	IQR	
Red blood cells	7.81	7.42–8.12	9.92	9.87–12.31	0.42
White blood cells	1.75	1.13–2.26	1.13	0.74–1.52	0.22
Platelets	1.00	0.63–1.47	2.12	1.41–2.36	0.10

**Table 2:** Leukocyte-poor platelet-rich plasma (LP-PRP) complete blood count (CBC) and transforming growth factor-beta 1 (TGF-β1) concentrations. Red blood cells were measured in M/mL, while white blood cells and platelets were measured in K/mL.

	Whole Blood		LP-PRP		*p* Value
	Median	IQR	Median	IQR	
Red blood cells	9.00	7.42–12.31	0.07	0.01–0.22	<0.01
White blood cells	1.33	0.96–2.22	0.77	0.29–1.88	0.20
Platelets	1.44	1.00–2.12	6.63	4.77–12.63	<0.01
TGF-β1, pg/mL	1630.5	1273.5–2677.0	18122.0	8577.5–27575.0	0.01

**Table 3:** Bone marrow aspirate comparison between control and losartan groups in complete blood count (CBC) values. Red blood cells were measured in M/uL, while white blood cells and platelets were measured in K/uL.

	Control		Losartan		*p*-Value
	Median	IQR	Median	IQR	
Red blood cells	3.97	3.94–4.09	4.32	4.16–4.77	0.15
White blood cells	8.85	7.98–10.02	8.49	6.82–11.83	0.99
Platelets	93.0	46.0–101.0	20.0	12.0–38.0	0.15

**Table 4:** Bone marrow concentrate (BMC) comparison between control and losartan groups in complete blood count (CBC) values. Red blood cells were measured in M/uL, while white blood cells and platelets were measured in K/uL.

	Control		Losartan		*p*-Value
	Median	IQR	Median	IQR	
Red blood cells	7.81	7.42–8.12	9.92	9.87–12.31	0.42
White blood cells	33.89	18.83–41.3	25.48	9.82–31.2	0.42
Platelets	55.0	36.0–69.0	15.0	9.0–29.0	0.04

**Table 5:** Manual extraction and centrifugation steps used to prepare leukocyte-poor platelet-rich plasma (LP-PRP) and bone marrow concentrate (BMC). Platelet-poor plasma (PPP).

Processing Steps for LP-PRP	Processing Steps for BMC
1. First spin: 800 RPM for 10 min	1. First spin: 1500 RPM for 10 min
2. Extract platelet-poor plasma (PPP) without disrupting “buffy coat layer”	2. Extract PPP and “buffy coat layer”
3. Hard spin: 3300 RPM for 6 min	3. Second spin: 3300 RPM for 6 min
4. Remove excess plasma to obtain 10% sample	4. Remove excess plasma discarded and extract “buffy coat layer” to obtain a 10% sample

## Abbreviations

BMA	Bone marrow aspirate
BMC	Bone marrow concentrate
CBC	Complete blood count
EndMT	Endothelial mesenchymal transition
IQR	Interquartile range
LP-PRP	Leukocyte-poor platelet-rich plasma
MSCs	Mesenchymal stem cells
PRP	Platelet-rich plasma
TGF-β1	Transforming growth factor-beta 1
WB	Whole blood
